# Germination temperature sensitivity differs between co‐occurring tree species and climate origins resulting in contrasting vulnerability to global warming

**DOI:** 10.1002/pei3.10108

**Published:** 2023-04-24

**Authors:** João C. Filipe, Collin C. Ahrens, Margaret Byrne, Giles Hardy, Paul D. Rymer

**Affiliations:** ^1^ Department of Biodiversity, Conservation and Attractions Biodiversity and Conservation Science Perth Western Australia Australia; ^2^ Centre for Terrestrial Ecosystem Science and Sustainability Harry Butler Institute Murdoch University Murdoch Western Australia Australia; ^3^ Hawkesbury Institute for the Environment Western Sydney University Richmond New South Wales Australia; ^4^ School of Biotechnology & Biomolecular Sciences University of New South Wales Sydney New South Wales Australia; ^5^ Research Centre for Ecosystem Resilience Royal Botanic Gardens and Domain Trust Sydney New South Wales Australia; ^6^ Cesar Australia Brunswick Victoria Australia

**Keywords:** climate change, conservation, forest ecology, life‐history traits, local adaptation, range shift, safety margin, seed germination, thermal niche

## Abstract

Climate change is shifting temperatures from historical patterns, globally impacting forest composition and resilience. Seed germination is temperature‐sensitive, making the persistence of populations and colonization of available habitats vulnerable to warming. This study assessed germination response to temperature in foundation trees in south‐western Australia's Mediterranean‐type climate forests (*Eucalyptus marginata* (jarrah) and *Corymbia calophylla* (marri)) to estimate the thermal niche and vulnerability among populations. Seeds from the species' entire distribution were collected from 12 co‐occurring populations. Germination thermal niche was investigated using a thermal gradient plate (5–40°C). Five constant temperatures between 9 and 33°C were used to test how the germination niche (1) differs between species, (2) varies among populations, and (3) relates to the climate of origin. Germination response differed among species; jarrah had a lower optimal temperature and thermal limit than marri (*T*_o_ 15.3°C, 21.2°C; ED_50_ 23.4°C, 31°C, respectively). The thermal limit for germination differed among populations within both species, yet only marri showed evidence for adaptation to thermal origins. While marri has the capacity for germination at higher thermal temperatures, jarrah is more vulnerable to global warming exceeding safety margins. This discrepancy is predicted to alter species distributions and forest composition in the future.

## Graphical abstract

Climate change is shifting temperatures from historical patterns. Seed germination is temperature‐sensitive, making the persistence of populations and colonization of available habitats vulnerable to warming. The thermal limit for germination differed between species and among populations. This discrepancy is predicted to alter species distributions and forest composition in the future.

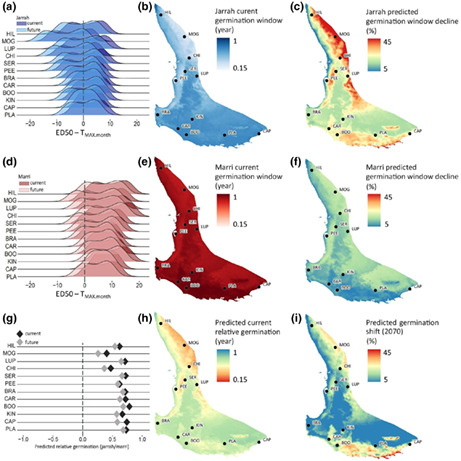

## 1 INTRODUCTION

Rapid anthropogenic climate change is decoupling organisms from their natural thermal ranges (Cheaib et al., 2012; Cobb et al., 2017). Temperatures will continue to change over the next 100 years and particularly exacerbated if emissions remain unchanged (IPCC, 2021). The dissociation between population‐specific adaptations and future temperature might cause reductions in population fitness (Kopp & Matuszewski, 2014; Ooi, 2012), shifts in species composition (Morin et al., 2018), range contractions (Jiménez‐Alfaro et al., 2014), and local extinctions (Wiens, 2016). Trees are particularly vulnerable, as they require hundreds to thousands of years for evolutionary change to occur (Lenoir et al., 2020; Lind et al., 2018). However, not all tree species and regions are equally vulnerable to changing temperatures due to their varying trait characteristics (Ahrens, Andrew, et al., 2019), contrasting evolutionary histories and adaptive capacity to climate change (Savolainen et al., 2007). Adaptive capacity is the combination of genetically determined species trait variation and the variation determined by environment (i.e., plasticity) to optimize the traits among variable conditions (Frankham, 2005). Data on intra‐ and interspecific variability are generally lacking, making it challenging to determine adaptive capacity within ecological communities and quantify vulnerability to future temperature conditions.

Assessing the vulnerability of organisms to changing temperatures is complex. The Intergovernmental Panel on Climate Change defines vulnerability as “the propensity or predisposition to be adversely affected by a threat, including sensitivity or susceptibility to harm and lack of capacity to cope and adapt” (IPCC, 2021), that is, it refers to the sensitivity of the impacted system to climate change along with the likelihood of exposure to harmful conditions. Most climate change impact studies use species distribution and niche modeling to infer vulnerable species and regions (Oddou‐Muratorio et al., 2020). However, vulnerability to warming depends on characteristics rarely included in these approaches, such as genetic variation, phenotypic plasticity, and life‐history traits (Aitken et al., 2008; Anderson et al., 2011; Christmas et al., 2016).

Life‐history traits that rely on temperature as an environmental cue are particularly at risk of negative impacts from anthropogenic global temperature changes (Saavedra et al., 2003; Walck et al., 2011). Indeed, temperature warming has been widely shown to limit the evolutionary potential of plant species, primarily through range contractions (Araújo et al., 2013; García‐Valdés et al., 2013), with significant downstream effects on community composition and structure, vegetation pattern, and functions of forest ecosystems (Alberto et al., 2013; Rong et al., 2019). To date, there is a lack of research assessing vulnerability to temperature variation that explicitly quantifies exposure and sensitivity to these shifts, assesses relative vulnerability of functionally important co‐occurring species, and identifies impacts on community composition and function.

Seed germination is one of the most critical life‐history stages for population persistence and colonization in a changing climate. Germination is primarily controlled by two environmental factors: temperature and water availability (Baskin & Baskin, 2014; Dürr et al., 2015). Water is generally required for seed germination, which is regulated by temperature to allow optimal levels within the species' thermal niche (Donohue et al., 2010). While extreme temperatures inhibit germination outside a temperature range to which the species is evolutionarily adapted (Baskin & Baskin, 2014), seeds of many plant species can generally germinate over a range of temperatures. Predicted increases in temperature will likely have a more significant effect on germination than later stages in the life history (Dalgleish et al., 2010; Walck et al., 2011), with even small changes in temperature considerably altering germination timing (Fernández‐Pascual et al., 2015; Mondoni et al., 2012), species richness (Lloret et al., 2004), and demography (Ooi, 2012).

The thermal niche of germination is described by an optimal temperature below and above which germination might be delayed or suppressed (Bewley et al., 2013). Indeed, there is a minimum (“base temperature,” *T*_b_) and a maximum temperature (“ceiling temperature,” *T*_c_) between which germination occurs, and an optimal temperature (*T*_o_) at which it occurs at the highest speed for the nondormant seed fraction under conditions of unlimited water availability, reflecting germination capability during seasons with available water (Dürr et al., 2015). Seed germination rate, a measure of seed vigor (Finch‐Sawage & Bassel, 2016), has an important relationship to temperature, which can be exploited to define cardinal temperatures for germination thermal range (Alvarado & Bradford, 2002; Rowse & Finch‐Savage, 2003). Thermal time‐to‐event modeling using cardinal temperatures provides a mechanistic comparison to describe thermal germination niche, which was initially developed on crop species (Covell et al., 1986; Ellis et al., 1986; Garcia‐Huidobro et al., 1982) and subsequently extended to wild species (Bloomberg et al., 2009; Midmore et al., 2015; Orrù et al., 2012). More recently, thermal time modeling has been applied to a limited number of wild species to define thermal safety margins and predict germination performance under various climate change scenarios (Fernández‐Pascual et al., 2015; Sampayo‐Maldonado et al., 2021; Seal et al., 2017). The germination thermal safety margin can be estimated as the difference between environmental temperature experienced by seeds and the upper and lower thermal limits within which germination can occur. If environmental temperatures continue to increase rapidly, the upper safety margins will become narrower and, if exceeded, the overall fitness, phenology, and timing of germination processes may be impacted (Cochrane, 2019). Therefore, germination temperature sensitivity is considered a significant indicator of vulnerability to climate change for some taxa (Cochrane, 2020; Cochrane et al., 2011).

The main objectives of this study were to explore germination thermal niche, adaptability and vulnerability to warming in two co‐occurring eucalypt tree species, *Eucalyptus marginata* Donnex.Sm. (jarrah) and *Corymbia calophylla* (R.Br.) K.D. Hill & L.A.S. Johnson (marri), endemic to the Mediterranean‐type climate of south‐western Australia. Broad‐scale tree canopy collapses have already been recorded in the Western Australia south‐west biodiversity hotspot, because of heatwaves and drought events (Matusick et al., 2013, 2018; Ruthrof et al., 2018) that are predicted to increase in intensity and frequency under climate change (IPCC, 2021). Understanding the sensitivity and adaptive capacity to warming temperatures is crucial to predict the vulnerability of species and populations and inform effective management plans and conservation strategies (Aitken & Bemmels, 2016; Keenan et al., 2015). Therefore, we aimed to define inter‐ and intraspecific variation in thermal thresholds for germination of both species by sampling 12 co‐occurring populations across their distribution. We tested the following hypotheses: (1) germination thermal niche will differ between species (species sensitivity), and marri will have higher thermal limits than jarrah, as jarrah has limited recruitment in the northern (warm) forests (Koch & Samsa, 2007; Norman & Koch, 2009); (2) germination responses will vary between populations (population sensitivity), where germination thermal limits will be positively correlated with maximum temperatures of origin (following genetic adaptation; Ahrens, Byrne, et al., 2019; Filipe et al., 2022); and (3) jarrah is more vulnerable to future temperature changes compared to marri, and northern (warm; Table 1) populations will be more vulnerable to future temperature conditions compared to southern (cool; Table 1) populations as a function of exposure to temperatures above the thermal limit for seed germination. By assessing the current thermal niche (sensitivity) and future windows (exposure) for germination, we quantified vulnerability to climate change at the species and population levels where the thermal safety margin of germination is exceeded. We found differences between species in their thermal limits and level of thermal adaptation. We predict vulnerability to global warming will lead to contraction of jarrah's range and altered forest composition with implications for forest conservation and restoration.

**TABLE 1:** Study locations and climate of origin of each sampled natural marri and jarrah population along with three climate variables for both the species distribution area. Mean annual temperature (°C; *T*_MA_), maximum temperature of the warmest month (°C; *T*_MAX_), and mean annual precipitation (mm; *P*_MA_).

Population	Code	Latitude	Longitude	Climate of origin	*T*_MA_ (°C)	*T*_MAX_ (°C)	*P*_MA_ (mm)
Hill River	HIL	−30.164	115.199	Warm and dry	19.6	33.9	558
Mogumber	MOG	−31.098	116.050	Warm and dry	18.7	33.5	554
Lupton	LUP	−32.529	116.500	Warm and dry	15.7	30.1	688
Chidlow	CHI	−31.862	116.226	Warm and wet	16.8	30.7	883
Serpentine	SER	−32.345	116.072	Warm and wet	16.3	29.7	1161
Peel Inlet	PEE	−32.692	115.710	Warm and wet	18.1	32.0	894
Bramley	BRA	−33.903	115.087	Cool and wet	16.6	27.6	1109
Carey	CAR	−34.425	115.822	Cool and wet	15.7	26.1	1164
Boorara	BOO	−34.612	116.206	Cool and wet	15.5	26.9	1194
Kingston	KIN	−34.082	116.337	Cool and dry	14.5	26.4	724
Cape Riche	CAP	−34.601	118.742	Cool and dry	16.1	25.7	585
Plantagenet	PLA	−34.653	117.499	Cool and dry	14.9	26.5	660

## 2 MATERIALS AND METHODS

### 2.1 Study species

**FIGURE 1:**
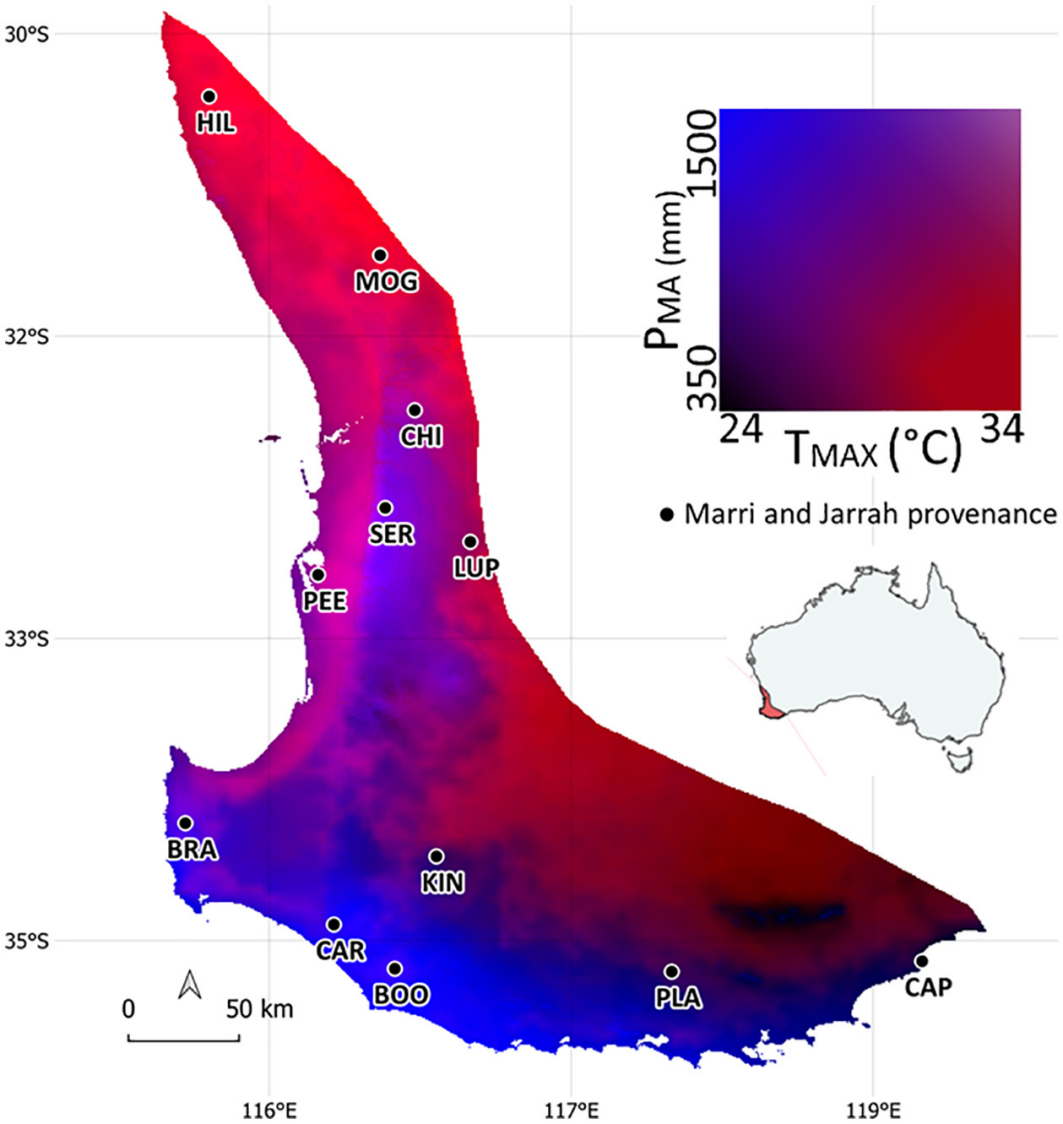
Sampled natural populations from marri and jarrah in south‐western Australia (black dots). Two climate gradients that can represent a limiting factor to species distributions are shown overlapped with color blending for the species distribution area. Maximum temperature of the warmest month (°C; *T*_MAX_ in the red display) and mean annual precipitation (mm; *P*_MA_ in the blue display). Insert shows the native distribution of marri and jarrah in Australia. Refer to Table 1 for the full populations' names.

*Eucalyptus marginata* (jarrah) and *Corymbia calophylla* Johnson (marri) are co‐occurring foundation tree species in the forests and woodlands of south‐western Australia (Whitford & Williams, 2002). They occur in areas with varying temperatures in the warmest month (*T*_MAX_) and mean annual rainfall (*P*_MA_; Figure 1). Jarrah seeds are released from mature and dried fruit capsules in the upper canopy (Christensen, 1971; Cremer, 1965), usually between December–March (summer to autumn). Jarrah seeds are viable between 4 and 4.5 mm (Cargill et al., 2019). Marri has a broad ecological distribution naturally occurring in a wide range of vegetation types, from tall forests in cool (southern) areas to open woodlands in warm (northern) regions. Marri fruits develop between March and December and release between January and February (summer; Johnstone & Kirkby, 1999). Marri seeds are usually 12–13 mm long (Boland et al., 1980; Johnstone & Kirkby, 1999).

### 2.2 Seed collection

Seeds from 120 individual mature trees from 12 natural populations were collected for each species across their co‐occurring geographic range (Figure 1; Table 1) during autumn–winter of 2019 (from March to August). Geographic coordinates were recorded for all sampled individuals using a handheld GPS device (Magellan eXplorist 310). The sampling, which covered an approximate area of 80,000 km^2^, included independent (>50 km separation) and replicate (across similar climate of origin) populations over both temperature and precipitation gradients. Sampled populations were grouped into four climate origins: Warm and dry, warm and wet, cool and wet, and cool and dry, according to *T*_MAX_ and *P*_MA_ of origin (“Warm” if *T*_MAX_ of origin >30°C, “Wet” if *P*_MA_ > 850 mm; Table 1). Mature capsules from each population were collected from 10 trees at least 100 m apart to reduce relatedness between individuals. Capsules were stored in silica gel until placed at room temperature (20 ± 1°C). Seeds were extracted by shaking capsules in closed sieves, separating most chaff from seeds. The number of seeds per capsule ranged from 1 to 7 for jarrah and from 3 to 4 for marri. Seeds damaged or predated by insect larvae were discarded. Whole plump seeds with no signs of discoloration were assumed to be viable (Cargill et al., 2019; Johnstone & Kirkby, 1999). After extraction, seeds were stored at 3°C, with 15% relative humidity in darkness until sowing.

### 2.3 Delineating minimum and maximum thermal response

This first experiment explored the minimum and maximum thermal limits of germination for both species using a bi‐directional temperature gradient plate (TGP; Model GRD1, Grant Instruments, Cambridge, UK; temperature range: +5 to 45°C). The germination responses were examined simultaneously over alternating and constant temperature gradients. The TGP allowed the exploration of germination responses to a complete thermal spectrum, allowing the delineation of appropriate temperature steps for the quantitative experiment (described below). For the TGP experiment, two populations were used, representing contrasting temperature and rainfall regimes: a cool temperature, high rainfall southern population (Boorara; BOO) and a warm, dry northern population (Hill River; HIL). Seeds were sown under sterile conditions in a laminar flow cabinet (metal‐free HWS; Clyde‐Apac) in 35 mm plastic Petri dishes on 0.75% w/v water agar with 10 seeds per dish for jarrah and 5 for marri, because of seed size differences. The moisture level was constant for all dishes, and dishes were sealed with parafilm to minimize moisture loss by evaporation. Dishes with seeds were placed into one TGP cell with a specific day/night temperature combination. Each of the four populations were exposed to all 49 temperature combinations, between 5 and 40°C, with a 12‐h photoperiod, that is, 5 to 40°C day‐time temperatures combined with 5 to 40°C night‐time temperatures with approximately 2.5°C increments among TGP cells (see Figure S1 for photos of sowed dishes and TGP setup). Temperature conditions cannot be replicated during an alternating bi‐directional run on the TGP, so each temperature condition was represented by only one dish. Seed germination was recorded three times weekly, and germinated seeds (a radicle double the length of the seed) were removed. At the end of the experiment, seed viability was tested by cutting non‐germinated seeds. Seeds with a hard, white endosperm were considered viable, all others were considered non‐viable. The TGP germination test ended when no new germinants were recorded for five consecutive days.

### 2.4 Delineating differences between populations and species

The second experiment was conducted using germination cabinet incubators (TRI‐145‐1‐SD; Thermoline Australia Pty Ltd.). The 12 sampled populations from each species were tested under five constant temperatures: 9, 15, 21, 27, and 33°C (based on the TGP experiment), with a 12 h day/12 h night photoperiod (Jackson, 2009). Individual chambers were used to test each temperature value, with temperature data loggers placed inside each chamber (top and bottom) to ensure constant and uniform temperatures across the chamber space. Seeds were sown in 100 mm plastic Petri dishes on 0.75% w/v water agar. Each dish contained 10 seeds from both species, to avoid biased effects in the data analyses. A total of three independent runs were performed. In each run, populations were represented by three dishes for each temperature treatment (30 seeds). A total of 9 replicates (dishes) from each species' population were tested for each temperature treatment, for a species total of 1080 seeds. The moisture level was constant and equal for all sowed dishes. Seed germination was recorded three times per week, with germinated seeds removed. At the end of the experiment, non‐germinated seeds were checked for viability with a cut test, and non‐viable seeds were removed from the original count. Each run ended when no new germinants were recorded for five consecutive days.

### 2.5 Data analysis and visualization

Experiment 1 data were visualized through contour plots showing percentage germination on the bi‐directional TGP. Contour plots were generated in R v4.1.1 (R Core Development Team, 2021) using the function *levelplot* from package “*lattice*” v0.20‐45 (Sarkar, 2008). Individual plots for each species‐population combination were created. Maximum mean germination percentage (*G*_MAX_) and time to 50% germination (*T*_50_) for each species were calculated from Experiment 2 data. *G*_MAX_ and *T*_50_ were calculated in R with the function *summaryBy* from the “*doBy*” v 4.6.11 package (Højsgaard et al., 2009).

For Experiment 2, the count data per day were transformed into time‐to‐event data (Onofri et al., 2011). A time‐to‐event model (TTEM) was applied with the *drm* function from the “*drcSeedGerm*” package (Onofri et al., 2018), setting the arguments “type” to “*event*” and “fct” to “*TTEM*”, which returned the following parameters: the optimal temperature for germination (*T*_o_), along with the upper and lower temperature for germination (*T*_c_, *T*_b_ respectively). To identify the temperature threshold for germination for each species population, the semi‐parametric method of the dose–response model (DRM) was applied (Ritz et al., 2019), using the function *drm* of the “*drc*” package (Ritz et al., 2015). A three‐parameter log‐logistic function (*LL.3*) was included, which considers the characteristic features of germination curves: repeated observations of the seeds over time, observation intervals, and lack of interdependence between proportions and variance homogeneity (Ritz et al., 2013). The DRM was fitted with a continuous curve, following the decline in germination after *T*_o_, to calculate estimated effective dose (ED) of temperature value to achieve 85%, 50%, and 15% of germinated seeds (i.e., germination rate) using the *ED* function (ED_85_, ED_50_, and ED_15_, respectively), along with 95% confidence intervals. The DRM was applied to all individual populations separately and again with each of the climate‐origin groups within each species (Table 1). The lowest temperature (9°C) data were removed from the DRM model for populations that had a significant decline in germination rate from the maximum germination rate, because we were only interested in the decline due to high thermal temperatures.

To explore how germination temperature has adapted to climate of origin, germination metrics for each marri and jarrah population were related to the climate at the seed source location. Current climate (1970–2000) temperature and precipitation layers for sampled populations were extracted from WorldClim v2 database (Fick & Hijmans, 2017) with a spatial resolution of approximately 1 km^2^, including annual mean temperature (*T*_MA_), the maximum temperature of the warmest month (*T*_MAX_), annual precipitation (*P*_MA_) and precipitation of warmest quarter (*P*_WQ_). Three of these represent variables (*T*_MAX_, *P*_MA_, and *P*_WQ_) which were shown to drive patterns of genetic adaptation in jarrah (Filipe et al., 2022); similarly, *T*_MAX_ and *P*_MA_ were also shown to be associated with genomic variation in marri (Ahrens, Byrne, et al., 2019). A linear model with ED_50_ as the response variable was used to test each climate predictor's significance (*T*_MA_, *T*_MAX_, *P*_MA_, and *P*_WQ_) separately using the function *lm* in R. The standard error (SE) of the estimated ED_50_ values was included as a weight argument for each data point in the subsequent linear regression analysis. The weighting accounts for the uncertainty in the estimated ED_50_ value and gives more weight to the data points with more precise estimates. Significant relationships (*p* < .05) were visualized using *ggplot2* (Wickham, 2009) in R.

A monthly temperature variable (monthly average maximum temperature; *T*_MAX.month_) was also extracted from WorldClim v.2 to define annual germination windows under the current climate. Future climate data (2070) for *T*_MAX.month_ from global climate model projections of BCC‐CSM2‐MR (Wu et al., 2019) were modeled to predict future germination windows. The annual germination window was defined as the number of months in a year during which the germination rate achieved 50% for each population per species combination. The thermal safety margin for germination was defined as ED_50_ − *T*_MAX.month_ > 0. Ridgeline plots for current and future germination window shifts were generated with *ggplot2* (Wickham, 2009) in R. Generalized additive models (GAM) were built in R with package “*mgvc*” (Wood, 2017) with current and future germination window (in months) as the response variables, and three current climate variables as predictors: *T*_MA_, *T*_MAX_ and mean temperature of the wettest quarter (*T*_MWQ_). The GAM results were interpolated with the function *predict* to map the current germination window and predicted germination window decline (2070) across the species distribution. Predicted current relative germination was calculated as: [jarrah's current germination window]/[marri's current germination window]; while the predicted germination shift was defined as: [jarrah's germination window decline] − [marri's germination window decline]. All maps were generated with QGIS v3.22.5 (QGIS.org, 2022).

## 3 RESULTS

### 3.1 Minimum and maximum thermal response

**FIGURE 2:**
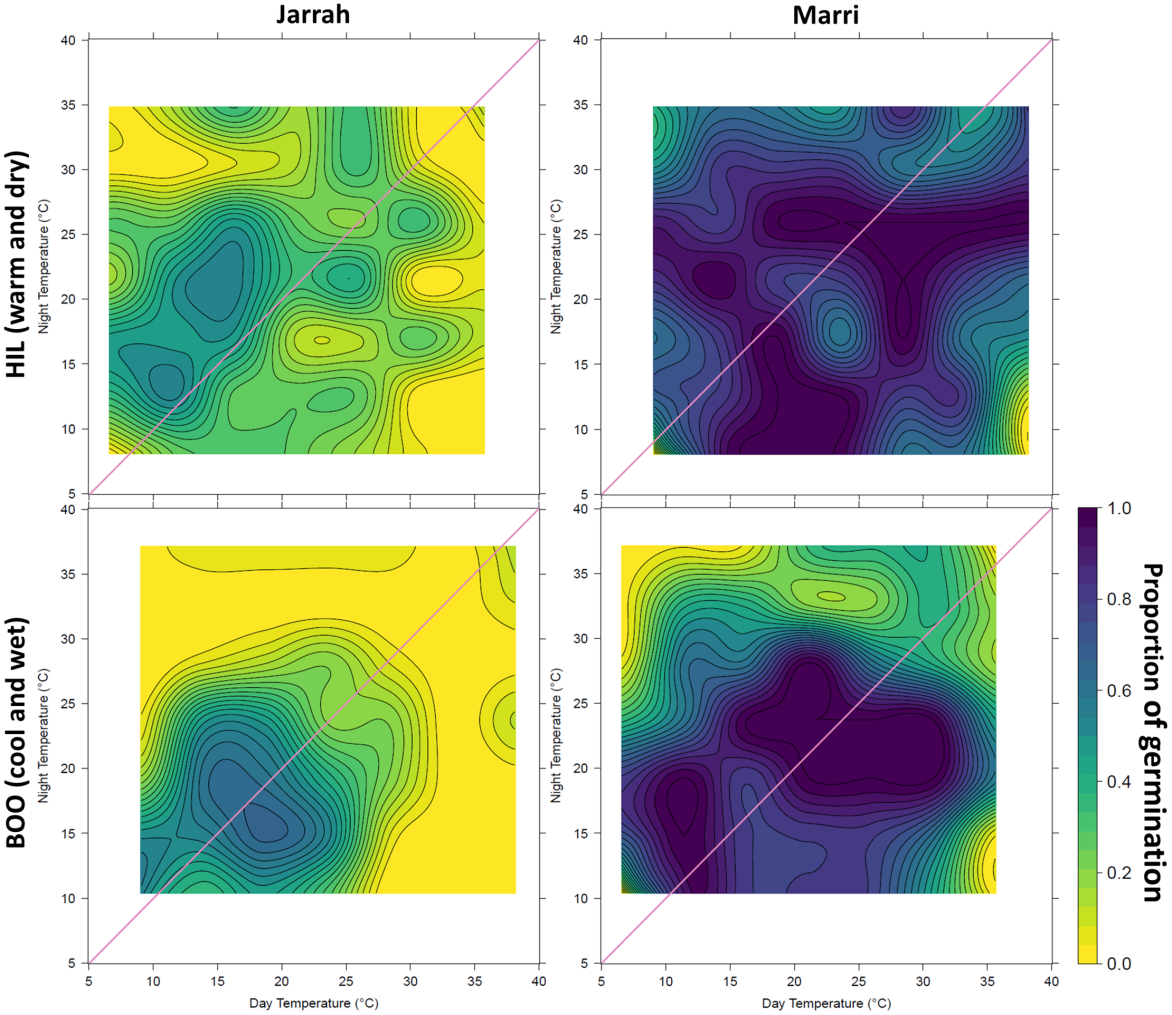
Bi‐directional temperature gradient plate contour plots for seeds from jarrah and marri populations of HIL (Hill River, warm and dry) and BOO (Boorara, cool and wet). The color gradation from dark purple (100%) to bright yellow (0%) represents the decreasing germination percentage. Isopleths connect points of equal percentage germination. Constant temperatures are presented on the diagonal line from the top‐right corner (maximum temperature 40°C) to the bottom‐left corner of the plots (lowest temperature 5°C). All points above and below the diagonal line represent alternating temperature regimes, with the greatest amplitude at each graph's top‐left and bottom‐right corners. The diagonal line from the bottom‐left to top‐right corner of each plot also signifies the divide between diurnal cycles that have light during the warmer day regime (bottom‐right section) and dark during the warmer day regime (top‐left section).

The TGP revealed high levels of seed viability (non‐viable seeds: <0.5% for jarrah, <1% for marri). There was visible germination variation for temperature and timing across the two species. The contour plots illustrate the species‐specific nature of seed responses to temperature (Figure 2). The time to first germination in marri was 2 days, while for jarrah first germination was recorded after 7 days; marri recorded no new germinants from Day 22 onwards, while jarrah germinated until Day 30.

Jarrah showed an overall low and constrained temperature window for germination. The total mean proportion of germination was 19%. The maximum proportion of germination was moderate and similar between populations: cool‐wet jarrah‐BOO at 66%, and warm‐dry jarrah‐HIL reached 53% (Table 1 for population details). Distinct response patterns were observable between species and populations across the plate (Figure 2). Jarrah‐BOO displayed an almost symmetrical shape of greater germination (25%–66%) in the bottom‐left corner, where temperatures were lower (closer to 5°C), with very low (<20%) or absent germination across the rest of the plate; while for jarrah‐HIL had higher germination rates in higher temperatures with several pockets of moderate–low germination (25%–35%) distributed randomly across the plate. In contrast, marri displayed several temperature combinations where plate cells reached 100% germination for both populations (Figure 2). The total mean germination proportion for marri was 64%. The warm‐dry marri‐HIL had high germination throughout except for a few pockets (>80%). High germination patterns for cool‐wet marri‐BOO is more isolated to cooler night temperatures with low germination (<25%) close to the top edge of the plate.

### 3.2 Cabinet incubators

**FIGURE 3:**
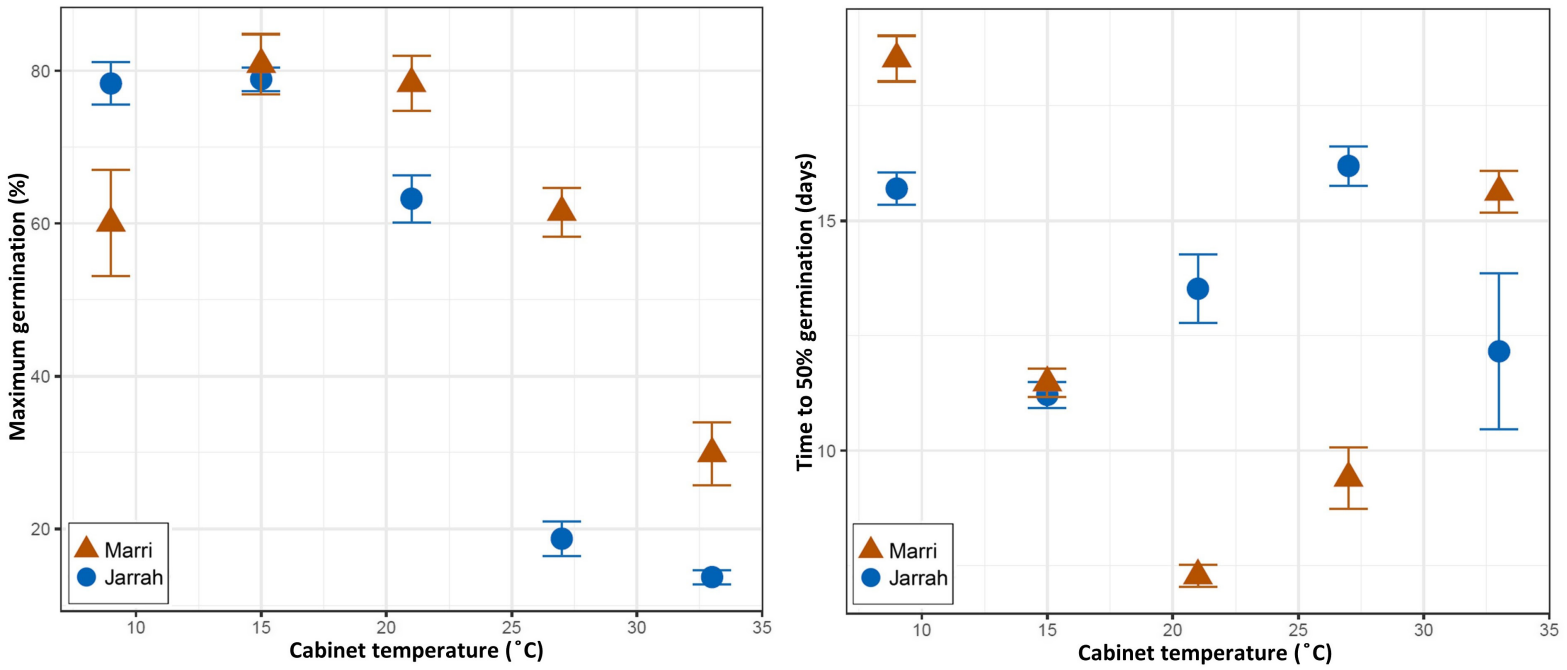
Mean maximum germination percentage (*G*_MAX_; left panel) and mean time to 50% germination in days (*T*_50_; right panel), with SE bars, for marri and jarrah exposed to constant temperatures for 35 days: 9, 15, 21, 27, and 33°C.

The overall results from the TGP trial indicate that a temperature range from 9 to 33°C would be sufficient to differentiate between species and populations. After each of the three runs, cut tests found a negligible proportion of non‐viable seeds (<1% for both species). The mean time (averaged from three runs) for first germination in jarrah was 6.5 days, and for marri was 7 days. Maximum mean germination percentage (*G*_MAX_) and mean time to 50% germination in days (*T*_50_) calculated for marri and jarrah (9 dishes per treatment for each species) revealed clear species' differences in the thermal niche (Figure 3). For the lowest temperature treatment (9°C), jarrah showed a high *G*_MAX_ (78%), while marri recorded a lower mean value (60%); when the temperature was increased to 15°C, both species showed a similarly high response (marri—82%; jarrah—79%). Although, at 21°C, the species were again separated; germination in jarrah drastically decreased from 21 to 27°C, shifting from 64% to 18% *G*_MAX_, until a record low was registered at 33°C, with 14% *G*_MAX_. *G*_MAX_ in marri was consistently higher than jarrah but with smaller drops in *G*_MAX_ between 21 and 33°C (70% to 30%). The *T*_50_ response for jarrah required less time to achieve 50% germination (*T*_50_ = 15.5 days), while marri only reached 50% germination after 18.5 days; similarly, to *G*_MAX_, at 15°C both species showed a similar response for *T*_50_, at around 11.5 days, and from 21°C onwards the species were separated, with jarrah scoring higher *T*_50_. At the highest temperature (33°C) marri recorded a higher *T*_50_.

### 3.3 Dose–response modeling

Jarrah recorded an overall 48% maximum seed germination, while marri had 61% seed germination. The optimal temperature (*T*_o_) for marri was higher (*T*_o_ = 21.2°C) than jarrah (*T*_o_ = 15.3°C). The germination response curve estimated with a log‐logistic dose–response model (DRM) revealed that all the ED parameters for marri (by the climate of origin groups and individual populations) scored higher temperature predictions compared to the parameters for jarrah (Table 2; Table S1). The highest ED_50_ was recorded for marri warm‐dry climate (33°C), followed by the warm‐wet (32.1°C), both higher than the estimated ED_50_ for the cool climates (29.4°C); for jarrah, ED_50_ shows less variation between climate groups (<1°C), ranging from 23.4°C (cool‐dry) to 24.4°C (warm‐dry). Notably, ED_50_ differed by >8.5°C between species for the warm climate groups.

**TABLE 2:** Dose–response model ED_85_, ED_50_, and ED_15_ estimates for the climate of origin population groups within marri and jarrah.

DRM prediction		85% germination	50% germination	15% germination
Species	Climate of origin	ED_85_ (SE)	ED_50_ (SE)	ED_15_ (SE)
Marri	Warm and dry	31.6 (1.7)	33.0 (0.3)	34.4 (1.8)
	Warm and wet	28.3 (1.5)	32.1 (0.7)	36.0 (1.2)
	Cool and wet	24.5 (0.7)	29.4 (0.5)	34.3 (0.7)
	Cool and dry	25.4 (0.8)	29.4 (0.5)	33.4 (0.8)
Jarrah	Warm and dry	19.9 (0.7)	24.4 (0.5)	29.9 (0.9)
	Warm and wet	20.2 (0.6)	23.5 (0.4)	27.3 (0.7)
	Cool and wet	19.9 (0.6)	23.7 (0.3)	28.4 (0.7)
	Cool and dry	20.5 (0.4)	23.4 (0.3)	26.8 (0.5)

**FIGURE 4:**
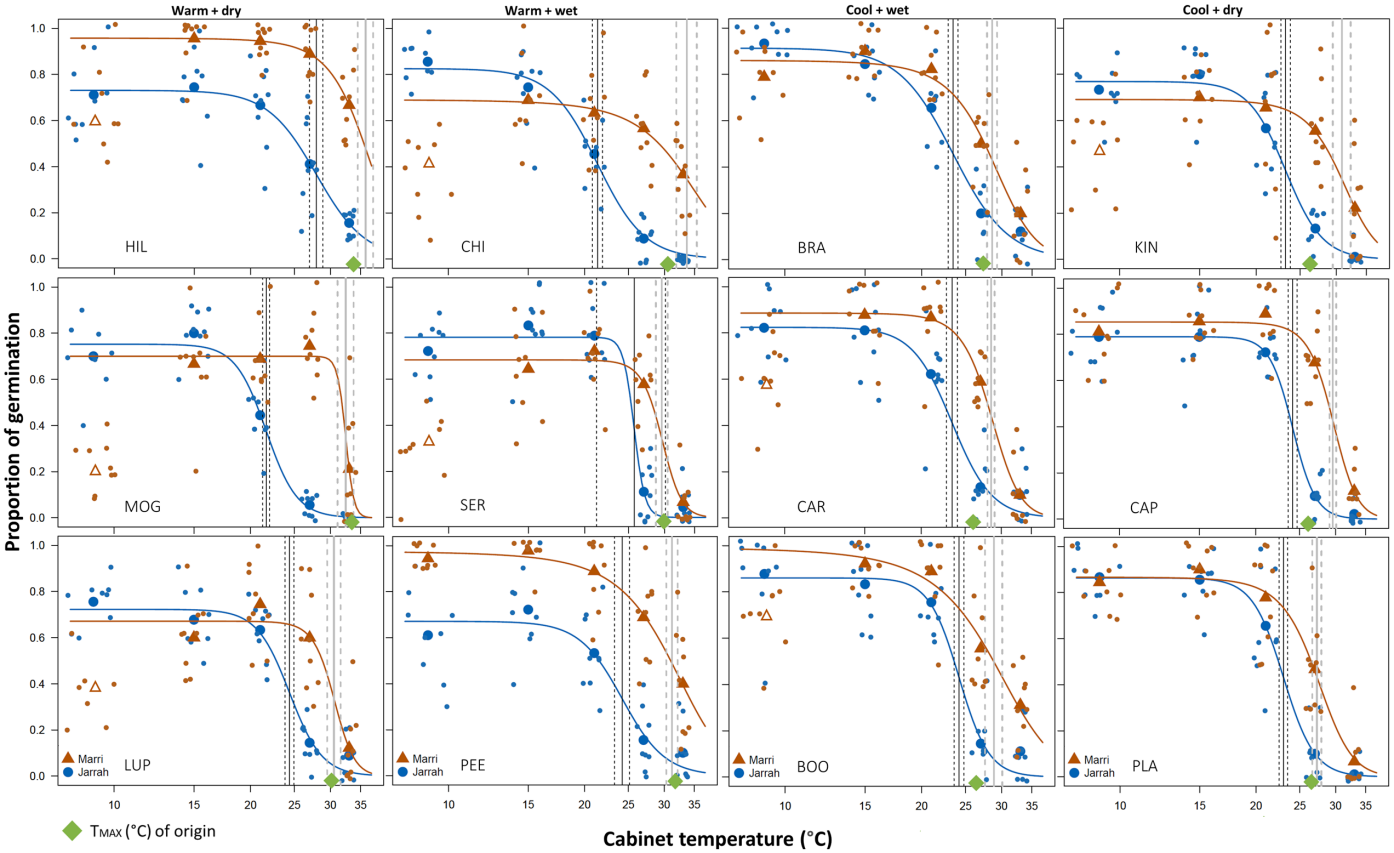
Germination response curves for marri and jarrah populations exposed to constant temperatures for 35 days: 9, 15, 21, 27, and 33°C. The climate of origin is labeled at the top of each column. Each small data point is the proportion of germination from individual dishes and the bigger data points are the population mean under each temperature dose. Vertical lines denote ED_50_ (± SE in dashed lines) for each marri (gray) and jarrah (black) population. A green diamond marks the mean maximum temperature of the warmest month (*T*_MAX_, °C) of origin for each population. Empty triangles denote marri populations for which the 9°C treatment was dropped from the model fit. Refer to Table 1 for the full populations' names.

Individual DRM curves were plotted to visualize population temperature responses comparing species (Figure 4). For most warm populations, jarrah recorded a higher proportion of germination than marri for the lower tier of temperatures (9 and 15°C). A swap between species occurred around 21°C, with germination depressed onwards for both species and marri keeping a higher germination proportion for the higher temperatures. The exceptions for this pattern are HIL (warm‐dry) and PEE (warm‐wet), for which marri scored a higher proportion of germination across the range of temperatures. The same three warm populations for both species (MOG, SER, and LUP) recorded an extremely low germination response (<5%) for the highest temperature treatment (33°C). For the cool climate populations, the variation between jarrah and marri was smaller or even overlapping (PLA, 9–15°C). Jarrah cold‐climate populations showed a higher germination response than marri at lower temperatures (9 and 15°C) for two populations (BRA and KIN), while marri cold populations consistently recorded a higher germination proportion than jarrah for higher temperatures (>21°C), similar to the patterns found across warm populations.

The temperature threshold for germination was estimated as ED_50_. Jarrah scored lower ED_50_ than marri in all co‐occurring populations (Table S1). While the jarrah population with the warmest climate of origin (HIL, *T*_MAX_ = 33.9°C) recorded the highest ED_50_ for the species (27.7 ± 1.1°C), the two most temperature‐sensitive jarrah populations also belong to the warm climate of origin (MOG, *T*_MAX_ = 33.5°C, ED_50_ = 21.3 ± 0.3°C; and CHI, *T*_MAX_ = 30.7°C, ED_50_ = 21.9 ± 0.5°C). Marri showed a much clearer signature of the climate of origin with two cool‐climate populations (PLA and BRA) showing the lowest ED_50_ (27.1 ± 0.8°C and 27.8 ± 0.8°C, respectively) and the warmest population HIL recorded the highest ED_50_ (35.9 ± 1.4°C).

**FIGURE 5:**
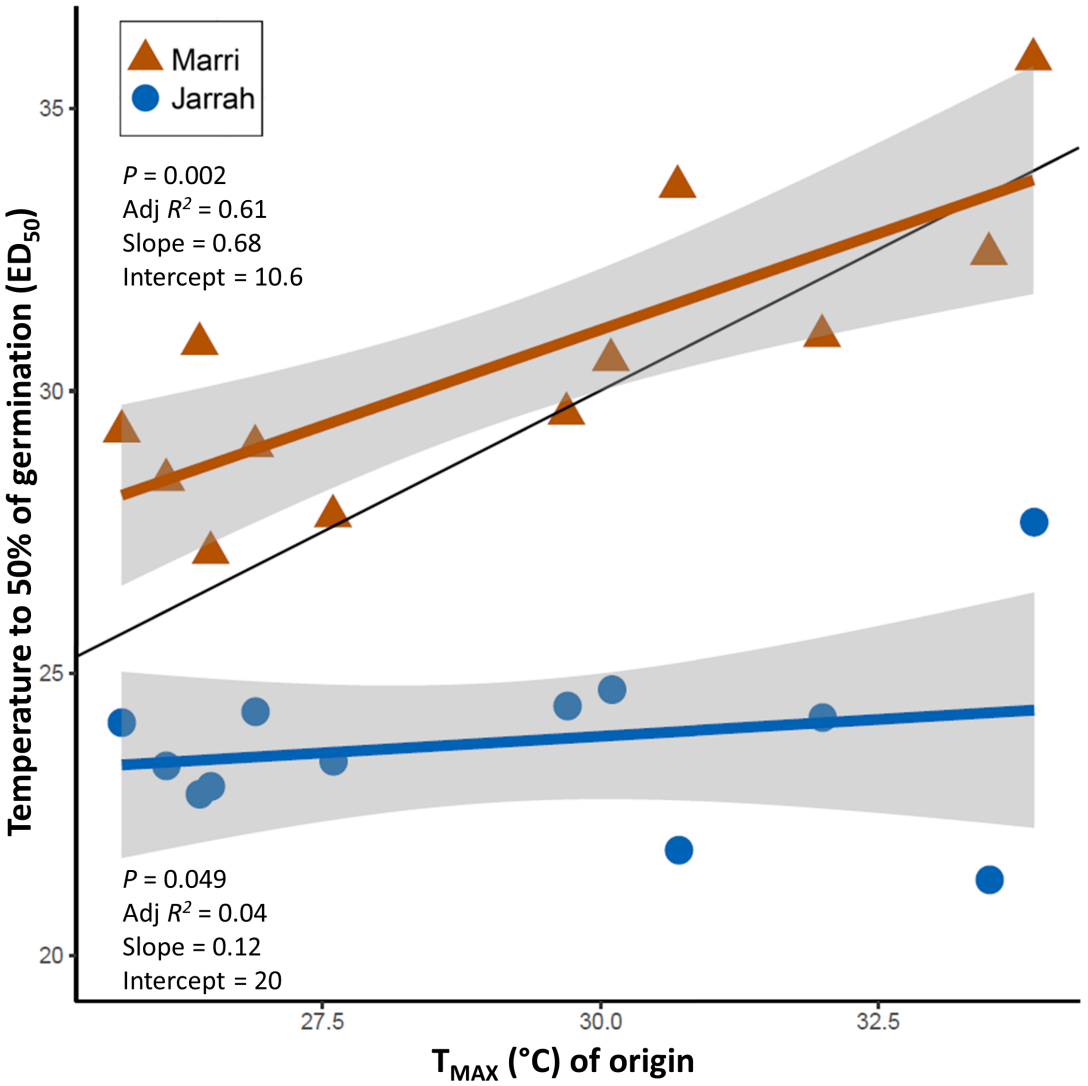
Correlation of the temperature required to reach 50% germination (ED_50_) against the mean maximum temperature of the warmest month (*T*_MAX_, °C) of origin for each marri and jarrah population. Identity line (*y* = *x*) in black.

The ED_50_ thermal threshold of germination was significantly predicted by the *T*_MAX_ of origin for marri and jarrah populations (*p* = .002, *R* ^2^ = .61; *p* = .049, *R* ^2^ = .04, respectively) (Figure 5). Marri showed a strong positive correlation where ED_50_ increased 0.68°C per degree Celsius in *T*_MAX_. Jarrah populations showed a weak relationship with a relatively flat slope (0.12) between *T*_MAX_ and ED_50_. Other climate metrics (*T*_MA_, *P*_MA_ and *P*_WQ_) were not significant predictors of ED_50_ among populations (*p* > .05).

*T*_MAX_ for sampled populations ranged from 25.7°C (CAP) to 33.9°C (HIL). Warm‐origin marri populations displayed ED_50_ estimates similar to *T*_MAX_; noticeable exceptions were HIL (ED_50_ of 35.8°C—*T*_MAX_ of 33.9°C) and CHI (ED_50_ of 33.6°C—*T*_MAX_ = 30.7°C) with higher (+1.9°C) thermal safety margins. Cool origin marri populations showed higher ED_50_ than respective *T*_MAX_, with the largest thermal safety margins found for KIN (+4.5°C) (Figure 4). All jarrah populations displayed ED_50_ values lower than the respective *T*_MAX_ such that the thermal safety margins were exceeded. However, the magnitude difference was greater for warm‐origin (>5°C) compared to cool‐origin populations (<4°C) (Figure 4).

### 3.4 Current and future germination window

**FIGURE 6:**
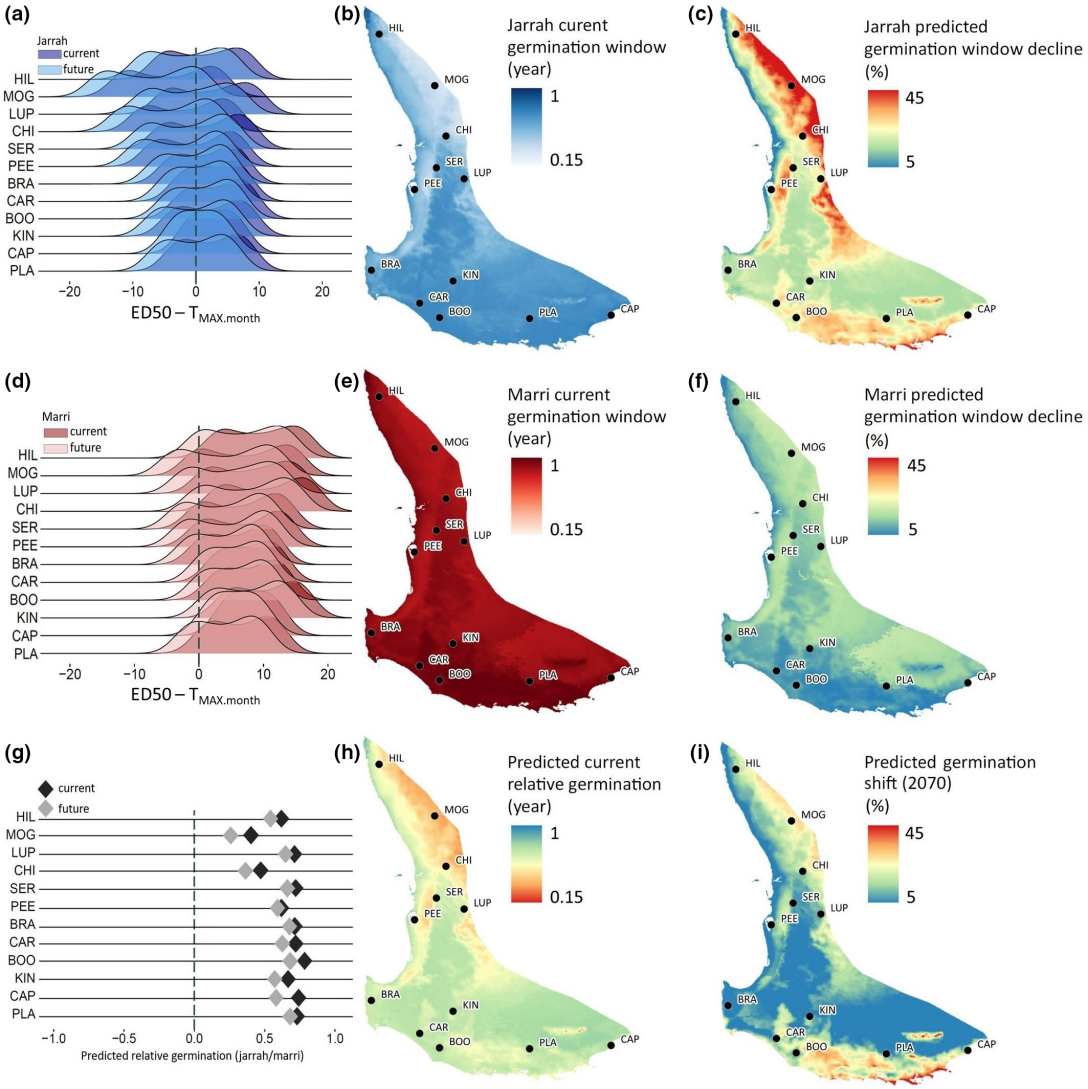
Thermal safety margin and predicted germination window under current and future (2070) temperatures for jarrah (*a*, *b*, and *c*, respectively) and marri (*d*, *e*, and *f*, respectively), along with relative germination among species (*g*, *h* and *i*) across the distribution. Density ridgeline plots showing current and future (2070) annual (12 months) germination window for each population from jarrah (*a*) and marri (*d*) with the vertical 0 lines indicating the thermal safety margin (ED_50_ − *T*_MAX.month_ = 0) and positive values for months for which germination occurs. The maps are based on generalized additive models (GAM) predicting the germination window based on climate metrics (Table S2). Refer to Table 1 for the full populations' names.

The annual germination window was predicted to decrease in the future (2070) for all sampled populations of jarrah and marri (Figure 6). As temperatures increase throughout the year the ED_50_ is exceeded by *T*_MAX_ in the warmer months reducing the number of months when germination is possible. All jarrah populations had a lower safety margin under current conditions compared to their marri counterparts resulting in a narrower germination window. The shortest current window for jarrah was found for population MOG (warm and dry): 4.1 months, which was also the population with the highest predicted decline for 2070: 2.3 months (43% decline), followed by CHI (warm and wet), with a 32% predicted drop (from 5.7 to 3.8 months); while the longest current windows for jarrah (>8.5 months) were found for three cool origin provenances (CAR, BOO and CAP). For marri populations, all current windows are >9 months, with MOG showing the lowest score (9.8 months), and at least six populations from diverse climate origins (HIL, CHI, CAR, BOO, KIN, and CAP) presenting a current window above 11.5 months. This indicates that germination rates of 50% would currently be possible for these marri populations.

Predictions of the GAM analysis (Table S2) provided interpolated maps of the species' thermal germination window over space and time (current and future; Figure 6). The current annual germination window for jarrah (min. = 0.34, in MOG; max. = 0.75, in CAP; Figure 6a,b) shows strong intraspecific variation within the northern (warmer origin) provenances, particularly from the coastal to eastern populations, and more uniform among southern (cooler origin) provenances. A similar pattern occurs for marri (min. = 0.83, in MOG; max. = 0.99, in CAP; Figure 6d,e), although with a noticeable area of shorter window as well in the south‐eastern (cool and dry) distribution. Consequently, predicted current relative germination highlighted the northern inland region (warm and dry) of the species distribution as the area with the lowest (<0.3) jarrah–marri germination ratio.

The effects of temperature warming predicted for 2070 were not uniform across species or populations. Jarrah showed an exceptionally steep decline in the predicted germination window (close to 45%; Figure 6c) around the northern inland range (warm and dry), the same region already with the shortest current window. Other pockets of intense decline (>40%) were also predicted in central (warm) provenances, both coastal (dry) and inland (wet), and in the south coastal region (cool and wet). The predicted window decline for marri was similarly the highest (20%–25%; Figure 6f) around the northern inland region, but also widely found for the south‐eastern area, following the matching trend with the already shorter current window. The lowest predicted current relative germination, which is indicative of the jarrah/marri germination ratio, displayed the lowest values (around 0.15%) in the northern region of MOG (Figure 6g). Interestingly, highest percentages (>35%) for the predicted germination shift (2070) were found for both northern inland (warm and dry) and south coastal (cool and wet) regions of the distribution (Figure 6h).

## 4 DISCUSSION

Climate change is leading to forest dieback events across the world, yet there is considerable uncertainty over the vulnerability of natural forests. Investigating the temperature constraints for germination can provide insights into the vulnerability of populations and species to climate warming. This study developed a multi‐species comparative framework, testing key hypotheses to explore climate adaptation in co‐occurring trees and inform forest management under climate change. We found significant inter‐ and intraspecific variation in the temperature response of germination for two co‐occurring species. However, only one species showed a high thermal safety margin and strong adaptive capacity with temperature of origin. We predict significant shifts in germination under a future climate scenario with shifts in forest species composition. Our approach provides a means of evaluating a key factor in vulnerability to future climate, and our findings will guide current and future management practices under climate change.

### 4.1 Temperature constraints for germination between species

Germination in jarrah was stimulated under cooler temperatures compared to marri. Confirming our first hypothesis, we found that jarrah had a lower optimal temperature where germination rates were relatively high under cool conditions compared to marri, where germination rates were more consistent across temperatures. Seed germination traits have been found to be significantly different among Mediterranean oaks (Amimi et al., 2020; Ganatsas & Tsakaldimi, 2013). However, these studies only sampled part of the species distributions, while here we sampled the whole distribution. Cochrane (2017) modeled observed germination data for 26 eucalypt species (including jarrah) to predict optimum germination responses (mean time to germination, germination timing and success) under current and future climate scenarios and found considerable interspecific variability among thermal germination thresholds. In another study, Catelotti et al. (2020) found that six *Persoonia* species had unique temperature sensitivities and optima profiles and reported that germination success was likely to decrease under predicted future climates. However, Cochrane (2017) used single populations, which induces a potential bias, while Catelotti et al. (2020) relied on TGP data only, which provides limited statistical quantification. Importantly, our work is consistent with these other studies in that germination rates differ among species yet builds on this by clearly demonstrating that co‐occurring species have developed different adaptive strategies to thermal cues.

Temperature is critical to defining the time and place for a seed to germinate (Baskin & Baskin, 2014; Gresta et al., 2010). Changes in the mean temperatures and the frequency and intensity of temperature extremes (e.g., heatwaves) can have a powerful impact on the achievement of early life‐cycle events. The lower temperature germination cue observed in jarrah is appropriate in the current climate because optimizing germination during cool temperature conditions is a key risk‐avoidance strategy against environmental stressors (e.g. drought), as it increases the chance of seedling survival under periods of favorable moisture conditions (Duncan et al., 2019). However, this strategy puts jarrah at risk of decline as winter temperatures increase. On the other hand, marri displayed the opposite trade‐off with better germination success in hotter environments, implying a strategy of broad germination across a range of temperatures to maximize recruitment. Ultimately, early germination may improve plant fitness but does not ensure effective establishment (Verdú & Traveset, 2005), and long‐term monitoring of recruitment in natural populations across climate gradients would be needed to validate whether predicted shifts in germination success equates to seedling recruitment and establishment.

### 4.2 Adaptive capacity to climate within populations

Physiological tolerance due to plasticity is an important attribute for species to track shifting climates (Jackson et al., 2009). Both study species germinated over a wide range of temperatures (c. 10–30°C), indicative of high plasticity. Eucalypts from temperate climates commonly germinate over wide temperature ranges, while extensive seed dormancy is more often observed for species from arid zones. For example, for species from temperate regions, *Corymbia maculata* and *Eucalyptus resinifera*, optimum temperature niche was reported as 13–28°C (Grant et al., 1997); while in *E. salmonophloia*, an endemic from central arid zones, optimum germination temperature niche is narrow (20–25°C; Yates et al., 1996). Phenotypic plasticity might allow plants to overcome adverse effects of temperature warming by rapidly adjusting traits to adverse conditions (Scheepens et al., 2018). Thus, flexible germination responses may allow populations to persist during rising temperatures (Clauss & Venable, 2000). Given that periodic extreme events such as heatwaves are expected to become more frequent, genotypes with enhanced plasticity in functional traits may be able to better sustain optimum performance relative to environmental variability compared to long‐term evolution of constitutive changes in mean traits (Alpert & Simms, 2002; Chevin & Lande, 2015; Gianoli & Valladares, 2012), particularly in environments with strong climatic variability (Scheepens et al., 2018). A rise in germination with increasing temperatures may be expected until threshold limit temperatures are exceeded.

Marri had a much greater adaptive capacity for high‐temperature germination threshold than jarrah (change in ED50 with *T*_MAX.month_), which supports our second hypothesis of intraspecific variation in germination responses. Various tree species show adaptive germination strategies to cope with climatic oscillations (Cochrane, 2020; Nicotra et al., 2010), and this adaptability may preserve co‐existence in forest communities under current climates (Turcotte & Levine, 2016). Another study looking at germination rate among single populations for 49 alpine species found germination to be strongly correlated with native habitat temperature (Rosbakh & Poschlod, 2015). Contrastingly, Cochrane (2020) found that the local climate of the seed origin did not drive seed responses in temperate species, nor was it indicative of temperatures for optimal germination. However, we found that climate‐origin significantly drove seed responses of populations for marri with a positive correlation between ED_50_ and *T*_MAX_, indicative of local adaptation to the thermal environment and the adaptive capacity to maintain germination success under higher thermal conditions. In contrast, jarrah had limited adaptive potential with little change in ED_50_ across the species distribution, except in the most extreme warm‐dry population (HIL). This pattern could be from isolation or greater selection pressure. While partial support for this hypothesis is provided by recent population genomic analyses (Filipe et al., 2022), further experimental work is required to determine the limits of adaptive capacity to warming.

Differences in seed size and quantity might explain variation in germination performance under warming temperatures. Marri seeds are approximately three times bigger than jarrah seeds (12–13 mm for marri, 4–4.5 mm for jarrah) and these co‐occurring species are disproportionally represented in transient seedbanks (5.3 jarrah seeds/m^2^ and 1.0 marri seeds/m^2^; Koch et al., 1996). Seeds from marri and jarrah do not show morphological dormancy (Baskin & Baskin, 2014); hence, viable seeds will not accumulate in the topsoil seedbank over inter‐seasonal periods (Cargill et al., 2019; Koch & Samsa, 2007). Previous findings show that seed size can respond to changes in climatic variables, including temperature (De Frenne et al., 2012; Soper Gorden et al., 2016), geographic gradients (Fenollosa et al., 2021; Murray et al., 2004), and changes in local factors such as water availability and soil pH (Fenner, 1992; Tautenhahn et al., 2008). Indeed, the trade‐off between seed size and quantity has been widely discussed, with larger seeds ensuring greater resource competitive ability and environmental tolerance, but lower dispersal ability and colonization capacity (Chen et al., 2019; Lebrija‐Trejos et al., 2016; Moles & Westoby, 2004). We hypothesize, therefore, that the seed size differences may contribute to the contrast in adaptive capacity among jarrah and marri. In support, some studies show that larger‐seeded species track better track climate change than small‐seeded species (Ash et al., 2017; Knight et al., 2020). We hypothesize that the larger seed size and (presumed) lower seed production observed for marri might be a potential trade‐off conferring enhanced thermal tolerance compared to jarrah.

### 4.3 Vulnerability to global warming

Exploring life‐history strategies to maintain fitness in co‐occurring species simultaneously provides unique opportunities to identify processes and patterns driving community assembly (Pearson et al., 2018), and the prospects for ongoing species coexistence (Gremer & Venable, 2014; Huebner et al., 2018). Previous single‐species studies exploring the thermal response of germination in trees have found that species distribution might not be affected by increased temperatures (Sampayo‐Maldonado et al., 2021). However, single‐species studies offer limited inferences regarding forest composition dynamics. Our comparative study shows that jarrah is more vulnerable to temperature change than marri on a seed‐to‐seed basis, due to its lower temperature threshold and less adaptive capacity to respond to warming across its natural distribution. Thus, the difference in germination rates between species could have a lasting effect on forest composition through changes in recruitment. This implies that levels of recruitment might be directly affected by total germination and germination rate (Cochrane, 2020). Recruitment constraints may then culminate in changed communities at local scales (Rosbakh et al., 2020; Tudela‐Isanta et al., 2018). In a study with two pine species, Ordoñez‐Salanueva et al. (2021) discuss the likely impact that intraspecific variation in phenotypic plasticity and fundamental thermal niches may have on species distributions under a changing climate, showing that population variation at the distribution margin will potentially affect species responses to a warming climate and will be significant in defining habitat suitability and species distributions under future climates.

High plasticity in germination traits found here, and in other functional traits (jarrah: Bleby et al., 2009; O'Brien et al., 2007; marri: Ahrens, Andrew, et al., 2019; Challis et al., 2022), may facilitate species' persistence under future warming. The ability to adjust physiological traits to changing environments through phenotypic plasticity has been extensively recorded in tree species (Prober et al., 2016) and shown to be heritable by subsequent generations (Matesanz et al., 2010; Nicotra et al., 2010). Indeed, evidence of phenotypic plasticity of germination traits in Mediterranean plants from the Northern Hemisphere suggests potential adaptation to a changing climate in the short‐ to medium‐term (Mattana et al., 2022). The application of threshold models to germination traits allows a more accurate approximation of the thermal effects on population vulnerability under future climate scenarios. The thermal requirements for germination provided in this study may be used to improve the ability of species distribution models to predict the persistence of these species under future scenarios because it explicitly measures sensitivity to future climates and consequently, vulnerability. Potential population decline, leading to altered vegetation community patterns (species composition and abundance), may coalesce into greater ecological impacts such as loss of function and increased biological invasions (Parolo & Rossi, 2008).

### 4.4 Applications

Conservation and management actions can be informed by a better understanding of species' temperature thresholds and adaptive capacity to respond to warming. We established the thermal safety margin and germination window for forest trees, based on a quantitative assessment of the temperature threshold of populations across climate of origin and estimated the vulnerability to future warming temperatures to show that species and populations will be differentially impacted. Despite observed declines in germination under high temperatures across all sampled populations, marri is not expected to suffer major germination depression due to predicted future (2070) temperature changes. In contrast, jarrah's current germination window is predicted to decline significantly in the next 50 years. The impact on germination rate will likely be greatest in jarrah's northern and western populations (warm and dry climate); however, impacts are also predicted in the central escarpment and southern populations. While the window of opportunity for germination may narrow with progressive warming projected for the region, particularly for jarrah, germination may still occur within a few months of favorable conditions (wet winters; Figures S2 and S3). Furthermore, inter‐annual climate variability may allow for periodic germination cooler years. Fluctuating germination rates may provide a bet‐hedging strategy (Venable, 2007) to improve the ability of tree species to persist in a changing climate (Simons, 2011); however, the continued pressure from warming combined with heatwaves may limit recruitment opportunities in the future (Harris et al., 2018).

Conservation efforts to maintain the persistence of warm and dry climate populations should look to introduce seed from the northern populations (e.g., HIL) into the more vulnerable, southern populations. Given that HIL is geographically and genetically isolated (Filipe et al., 2022) natural dispersal may be limited and active management strategies such as assisted gene migration may be needed (Aitken & Bemmels, 2016; Prober et al., 2015), but the potential for natural regeneration under warmer and drier climatic conditions has been understudied and requires more experimental investigations. Furthermore, ex situ conservation should complement in situ conservation approaches (Guerrant et al., 2004), including seed banking to secure genetic diversity for future conservation and restoration efforts (Walters, 2015). While marri populations may have enhanced capacity to persist in situ without active conservation management, thermal adaptation in germination (among other functional and physiological traits; Ahrens, Andrew, et al., 2019; Ahrens et al., 2021) provides a valuable genetic resource for restoration and forestry aiming to establish diversity and resilient forests for the future. These findings provide an evidence base for design and implementation of forest management strategies through the application of assisted gene migration and seed banking for restoration and forestry in the future.

## 5 CONCLUSIONS

We identified patterns consistent with divergent evolutionary strategies among co‐occurring foundation trees at the early life‐history stage of seed germination, which is critical for maintaining and establishing populations, with contrasting thermal thresholds and adaptive capacity to cope with global warming. Forest response to climatic change is a complex multiscale process, which is challenging to model and predict because of variability between species and populations. For seed germination, our study highlights greater vulnerability to warming in jarrah, notably in warm and dry climate populations, while marri might have sufficient thermal buffering capacity for germination to respond to anticipated global warming. The differential temperature response of germination for these co‐occurring species is predicted to lead to a fundamental change in the forests' composition over time. Given these trees are foundation species for the forest ecosystem, the change in composition is likely to have direct and indirect impacts on other plant and animal species across trophic levels with implications for maintenance of biodiversity, ecosystem function and resilience to current and future stressors. However, to validate these predictions, field surveys of species' relative abundance and recruitment across climate gradients are needed over time. Information on likely changes in ecosystem composition is required to guide forest management and restoration efforts worldwide.

## CONFLICT OF INTEREST STATEMENT

The authors declare no conflict of interest.

## Supporting information


Supporting Information S1

## Data Availability

The data supporting this study's findings will be openly available via a publicly available data repository upon acceptance.
