# Supplementary material for: Germination temperature sensitivity differs between co‐occurring tree species and climate origins resulting in contrasting vulnerability to global warming
Source: Plant Environ Interact. 2023 Apr 24;4(3):146–62. doi: 10.1002/pei3.10108 (PMC10290426; doi:10.1002/pei3.10108)
Supplement: Supplementary file 1 — Supporting Information S1 [file PEI3-4-146-s001.docx]

**Germination temperature sensitivity differs between co-occurring tree species and climate origins resulting in contrasting vulnerability to global warming**

**SUPPORTING MATERIAL**


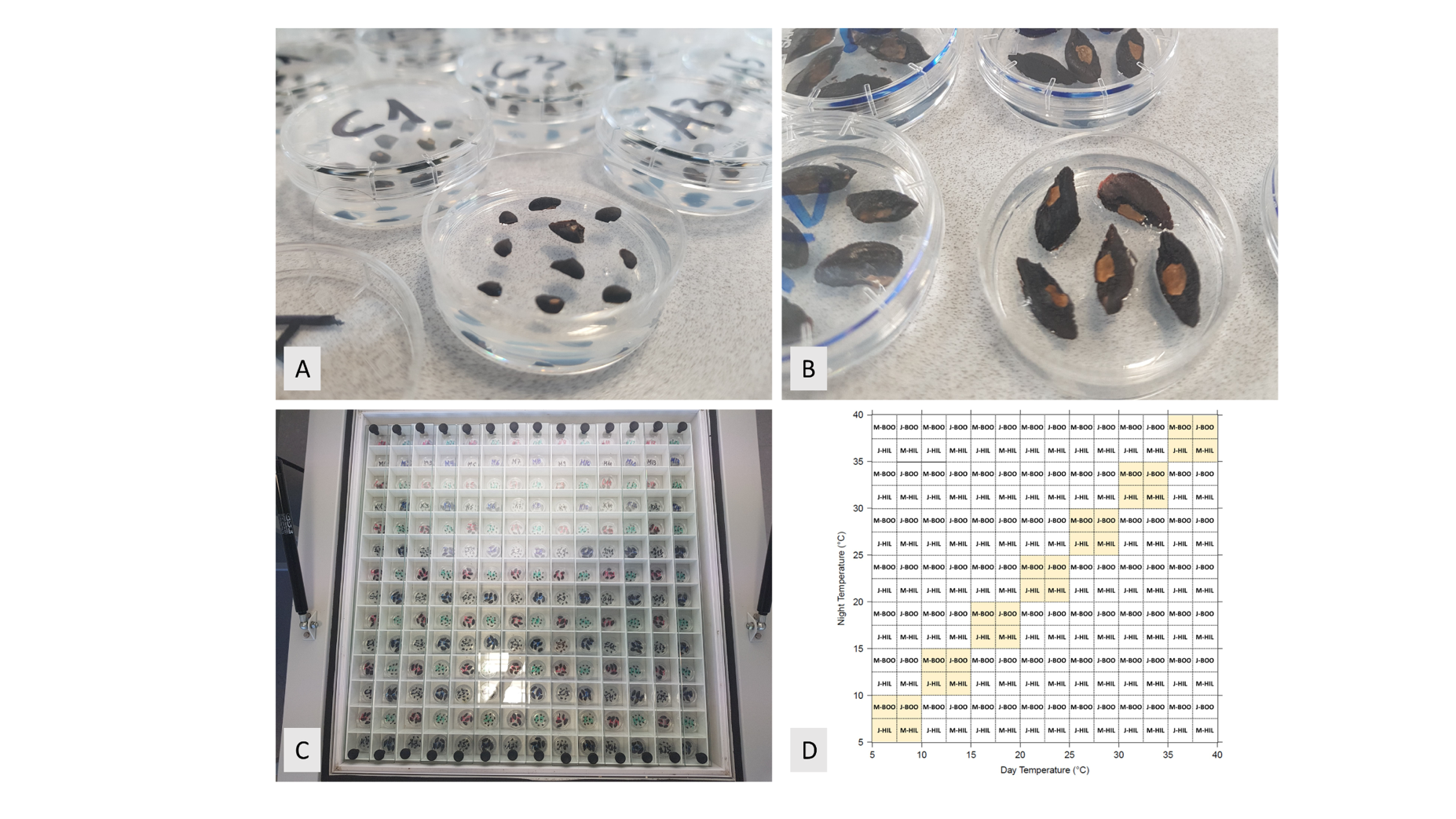


**Figure S1.** Seeds sown in 35mm plastic Petri dishes on 0.75% w/v water agar for jarrah (A) and marri (B), temperature gradient plate setup (C), and schematic experimental design (D). J – jarrah, M – marri, HIL - Hill River, BOO – Boorara. Constant temperatures are presented on yellow-shaded cells from the bottom-left corner of the plate (lowest temperature c. 5°C) to the top-right corner (maximum temperature c. 40°C).

**Table S1.** Dose-response model ED_85_, ED_50_ and ED_15_ estimates for each population of marri and jarrah.

| **DRM prediction** | |  | **85% germination** | | **50% germination** | | **15% germination** | |
| --- | --- | --- | --- | --- | --- | --- | --- | --- |
| **Species** | **Climate of origin** | **Population** | **ED_85_ (SE)** | | **ED_50_ (SE)** | | **ED_15_ (SE)** | |
| Marri | Warm and dry | HIL | 29.9 | (1.7) | 35.9 | (1.4) | 41.9 | (4.0) |
|  |  | MOG | 31.3 | (4.0) | 32.4 | (1.3) | 33.6 | (1.4) |
|  |  | LUP | 27.8 | (1.7) | 30.6 | (1.0) | 33.4 | (1.1) |
|  | Warm and wet | CHI | 25.6 | (5.4) | 33.6 | (1.7) | 41.7 | (5.9) |
|  |  | SER | 27.0 | (1.1) | 29.6 | (0.9) | 32.2 | (1.2) |
|  |  | PEE | 23.1 | (2.5) | 31.0 | (1.2) | 41.6 | (3.8) |
|  | Cool and wet | KIN | 26.2 | (2.6) | 30.8 | (1.4) | 36.3 | (2.8) |
|  |  | CAP | 26.2 | (0.6) | 29.3 | (0.5) | 32.8 | (0.8) |
|  |  | PLA | 23.1 | (1.8) | 27.1 | (0.8) | 31.9 | (1.4) |
|  | Cool and dry | BRA | 22.1 | (1.4) | 27.8 | (0.8) | 35.0 | (1.5) |
|  |  | CAR | 25.1 | (0.7) | 28.4 | (0.5) | 32.2 | (1.0) |
|  |  | BOO | 22.1 | (1.8) | 29.0 | (1.0) | 38.1 | (2.4) |
| Jarrah | Warm and dry | HIL | 22.0 | (2.0) | 27.7 | (1.1) | 34.8 | (2.1) |
|  |  | MOG | 18.3 | (0.7) | 21.3 | (0.3) | 24.9 | (0.8) |
|  |  | LUP | 21.7 | (1.1) | 24.7 | (0.6) | 28.1 | (0.8) |
|  | Warm and wet | CHI | 18.3 | (0.9) | 21.9 | (0.5) | 26.1 | (1.1) |
|  |  | SER | 22.2 | (1.2) | 24.4 | (0.8) | 26.8 | (0.6) |
|  |  | PEE | 20.0 | (1.3) | 24.2 | (0.9) | 29.3 | (1.8) |
|  | Cool and wet | KIN | 19.2 | (0.9) | 22.9 | (0.6) | 27.2 | (1.0) |
|  |  | CAP | 21.8 | (0.7) | 24.1 | (0.5) | 26.7 | (0.6) |
|  |  | PLA | 20.0 | (0.6) | 23.0 | (0.5) | 26.4 | (0.9) |
|  | Cool and dry | BRA | 18.4 | (1.0) | 23.4 | (0.6) | 29.8 | (1.2) |
|  |  | CAR | 19.6 | (1.2) | 23.4 | (0.7) | 27.9 | (1.4) |
|  |  | BOO | 21.4 | (1.0) | 24.3 | (0.7) | 27.6 | (0.9) |

**Table S2.** Generalised additive models (GAM) results in the relationship between current germination window and germination window decline (for each species), current relative germination and predicted germination shift, and three climate variables as predictors: mean annual temperature (T_MA_), maximum temperature of the warmest month (T_MAX_) and mean temperature of the wettest quarter (T_MWQ_).

|  |  |  | | | **Climate variable** | | | | | |
| --- | --- | --- | --- | --- | --- | --- | --- | --- | --- | --- |
|  |  |  | **T_MA_** | | | **T_MAX_** | | **T_MWQ_** | | |
| **GAM** | **Adj. R^2^** | ***p*-value** | **EDF*** | ***p*-value** | | **EDF** | ***p*-value** | **EDF** | ***p*-value** | **Dev. Expl.^#^** |
| Jarrah current germination window | 0.973 | 1.84 E-07 | 2.986 | 0.007 | | 1.954 | 0.038 | 2.522 | 0.031 | 99.1% |
| Jarrah germination window decline | 0.811 | 6.63 E-06 | 2.638 | 0.001 | | 2.574 | 0.047 | 1.518 | 0.004 | 92.7% |
| Marri current germination window | 0.313 | 7.92 E-11 | 1.000 | 0.002 | | 1.143 | 0.012 | 2.088 | 0.001 | 57.7% |
| Marri germination window decline | 0.585 | 1.10 E-06 | 1.000 | 0.044 | | 1.000 | 0.012 | 2.210 | 0.012 | 74.4% |
| Current relative germination | 0.834 | 1.66 E-07 | 2.864 | 0.008 | | 2.102 | 0.027 | 1.452 | 0.021 | 93.1% |
| Predicted germination shift | 0.630 | 2.00 E-04 | 2.472 | 0.016 | | 2.525 | 0.046 | 1.000 | 0.013 | 83.2% |

* EDF is the estimated degrees of freedom accounting for the smoothing function.

# Deviance explained by the model with three factors (i.e., climate variables).


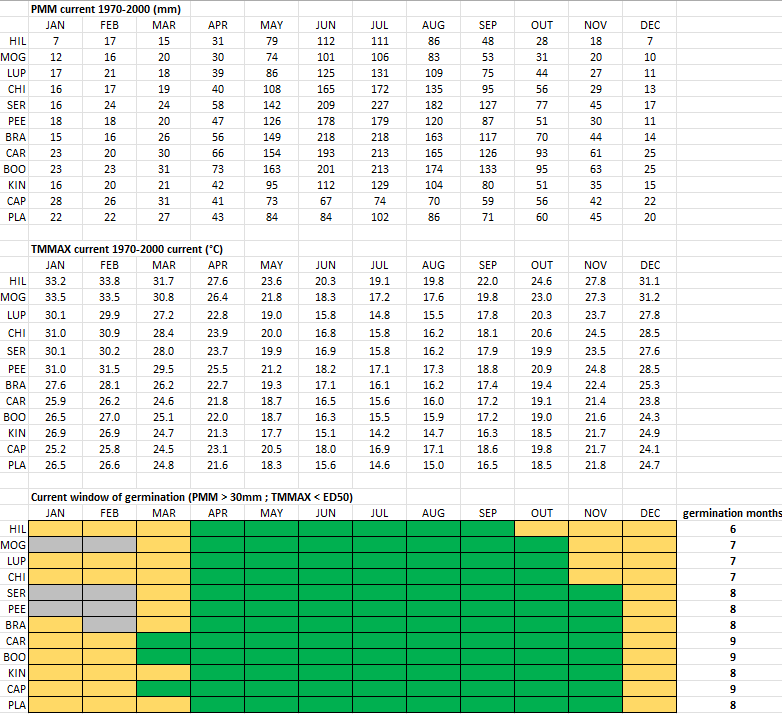


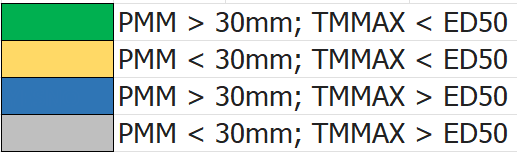

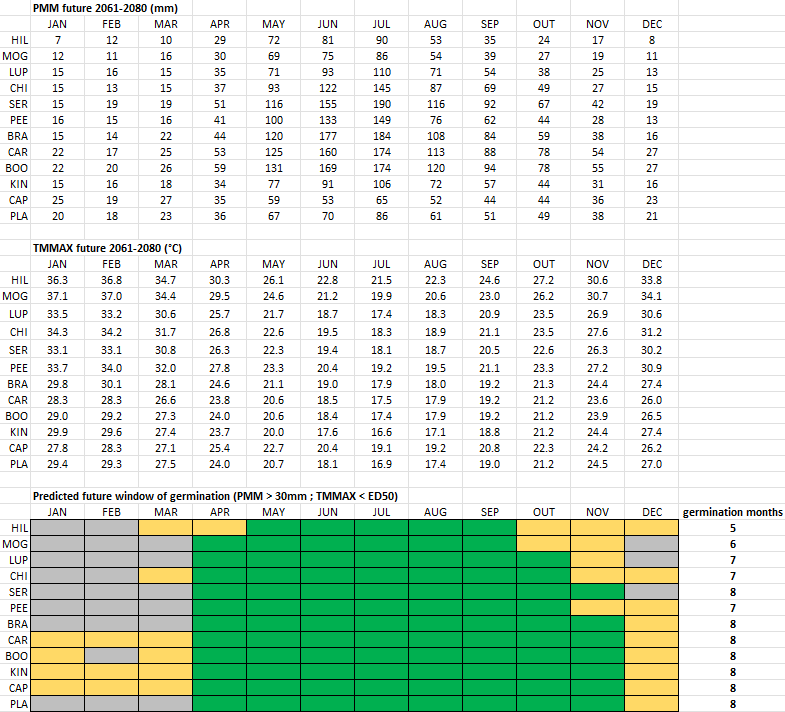


**Figure S2.** Current (top panel) and future (bottom panel) germination window for marri defined with climate thresholds of monthly total precipitation (PMM) >/< 30mm; and monthly average maximum temperature (TMMAX) >/< ED50. Green: germination occurs; Yellow: germination is limited by precipitation; Blue: germination is limited by temperature; Grey: germination is limited by both precipitation and temperature


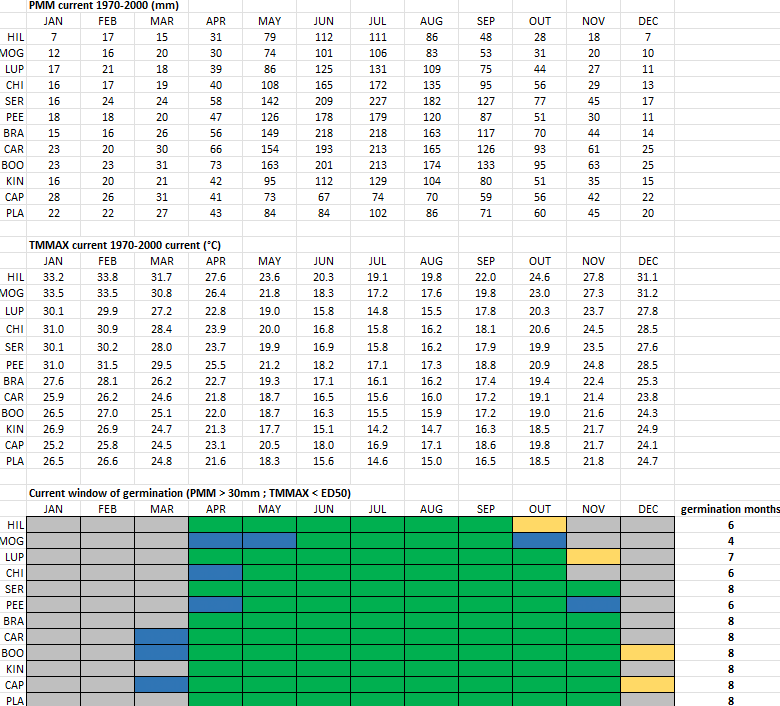


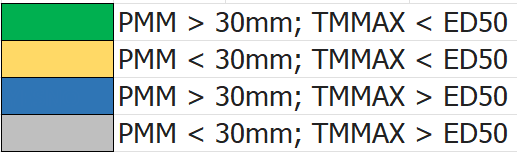

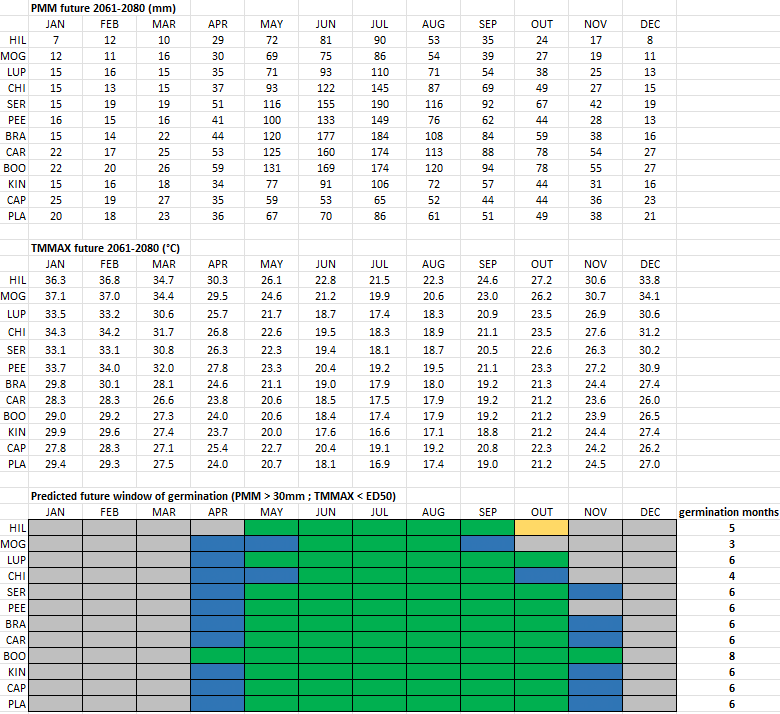


**Figure S3.** Current (top panel) and future (bottom panel) germination window for jarrah defined with climate thresholds of monthly total precipitation (PMM) >/< 30mm; and monthly average maximum temperature (TMMAX) >/< ED50. Green: germination occurs; Yellow: germination is limited by precipitation; Blue: germination is limited by temperature; Grey: germination is limited by precipitation and temperature
